# Schoolteachers for Action Against HIV/AIDS-related Oral Disease of Children in Africa

**DOI:** 10.3290/j.ohpd.b2805333

**Published:** 2022-03-14

**Authors:** Poul Erik Petersen, Febronia Kahabuka, Seydou Ouattara

**Affiliations:** a Professor, Institute of Odontology, Global Oral Health and Community Dentistry, Centre for Health and Society, Faculty of Health and Medical Sciences, University of Copenhagen, Denmark. Conceptualisation, data analysis, wrote original draft.; b Professor, Department of Orthodontics, Paedodontics and Community Dentistry, School of Dentistry, Muhimbili University of Health and Allied Sciences, Dar-es-Salaam, Tanzania. Data collection, review, editing.; c Senior Public Health Researcher, Health Systems Management Program, Centre MURAZ, National Institute of Public Health (INSP), Bobo-Dioulasso, Burkina Faso. Data collection, review, editing.

**Keywords:** HIV/AIDS oral lesions, oral disease prevention, schoolteachers, public health

## Abstract

**Purpose:**

The intention of the study was to evaluate whether Sub-Saharan schoolteachers may play a key role in oral health promotion of primary schoolchildren, particularly in terms of prevention of HIV/AIDS-related oral health problems.

**Materials and Methods:**

The study examined the level of knowledge about oral disease and the prevention of HIV/AIDS oral lesions amongst schoolteachers of Tanzania and Burkina Faso, identified their sources of information, and evaluated their ability for HIV/AIDS intervention. A multi-centre cross-sectional study was implemented in the two Sub-Saharan African countries. Participants comprised primary school teachers selected by stratified cluster sampling: 261 teachers from Tanzania and 313 teachers from Burkina Faso. All participants completed a structured questionnaire prepared for self-administration. The questions were designed from a standardised questionnaire developed by the World Health Organization.

**Results:**

Most teachers knew about the principal causes of the major oral diseases and the means of disease prevention. Nearly all teachers (95.6%) were aware of HIV/AIDS and they knew (92.3%) a virus was the direct cause of AIDS. Teachers were well informed of the general symptoms of HIV/AIDS, although oral symptoms were mentioned less often. In all, 17.6% of schoolteachers reported that children suffering from HIV/AIDS were found in their classes and 10.3% of teachers were conscious of students receiving anti-retroviral therapy (ART). Knowledge about the disease seemed to reflect mass media as a source, while teacher colleagues and health personnel played a somewhat lesser role in communication. In total, 83.2% emphasised that they should teach children about HIV/AIDS and the mouth. Schoolteachers from Tanzania (70.5%) were more often engaged in classroom-based oral health education than were the Burkinabe teachers (53.9%).

**Conclusion:**

The study confirms that schoolteachers may be a relevant source in the fight against HIV/AIDS among children. However, they would benefit from interaction with health personnel.

The suffering caused by HIV and acquired immunodeficiency syndrome (AIDS) remains an enormous human, social and economic burden, especially in low and middle income countries (LMICs), which account for the majority of all new infections.^20^ Meanwhile, combined efforts of numerous international, national and local partners imply that the number of people infected with HIV has continuously declined in many countries. In 2020, 37.7 million people globally were living with HIV, with 1.7 million children affected children.^22^ Currently, there are an estimated 20.6 million adults and children living with HIV in Eastern and Southern Africa, while the number for Western and Central Africa is 4.7 million people. Due to improved access to anti-retroviral therapy (ART), the number of People Living with HIV/AIDS (PLWHA) is today higher than ever. Worldwide, 27.5 million people live with HIV under ART, up from 7.8 million in 2010. In Eastern and Southern Africa, ART covers 57% of the children living with HIV, compared to 35% in Western and Central Africa.^6,22,23^

Globally, dental caries and periodontal disease are the two most common oral diseases in children. Dental caries is found in a national range of 33%-97% of children in LMICs, while approximately 90% show signs of mild or severe periodontal disease.^5^ Poor oral health and HIV/AIDS-related oral manifestations are often among the first symptoms of HIV/AIDS, and thus can be useful in early detection of the disease. In the oral cavity, HIV/AIDS may particularly manifest itself in the development of oral candidiasis, hairy leukoplakia, HIV periodontitis, oral ulcers, non-tender bilateral swelling of the parotid glands, and nodular tumours or Kaposi’s sarcoma.^2,3,13^ Moreover, HIV/AIDS infection may aggravate dental caries and periodontal disease.^4,5,8,14^ HIV specific lesions tend to decline with the use of highly active antiretroviral therapy (HAART), however, certain manifestations – mainly oral warts, parotid enlargement, and at some point oral squamous cell carcinoma – tend to increase.^7,15^

Previous studies have confirmed that Sub-Saharan African children frequently suffer from oral conditions related to HIV/AIDS, and poor oral health may often affect the daily life of children and school attendance in a negative way.^12,18^ As emphasised by the World Health Organization (WHO),^16^ information about HIV/AIDS-related oral manifestations and knowledge about disease prevention and self-care are therefore of greatest importance to the improvement of overall health and quality of life among PLWHA. However, a 2005 survey undertaken in Tanzania revealed limited knowledge about HIV/AIDS-related oral manifestations among PLWHA.^11^

Dental health workers are rare in most LMICs, with population to dentist ratios as high as 270,000:1 in countries like Tanzania and Burkina Faso.^17^ It is therefore necessary to use channels beyond the dental sector to create awareness of HIV/AIDS-related oral symptoms and to encourage self-care and healthy lifestyles. It is widely acknowledged that schools provide an ideal platform for the implementation of health promotion and population-directed disease prevention in children and families.^10,25^ Furthermore, primary schools are known as excellent settings for health promotion, reaching students, teachers and the wider community.

The school setting is used for HIV/AIDS-related interventions in several LMICs. Particularly, research has shown that the vulnerability of girls and young women to becoming infected with HIV may be reduced by improving HIV-related knowledge, skills and behaviour, and by refining their ability to act on HIV prevention messages.^9^ The WHO has recognised this by creation of the Health Promoting School concept, and a series of WHO documents has been developed on how to approach a number of health topics through schools, including HIV/AIDS^26^ and oral health.^27^

The intention of the present study was to highlight whether Sub-Saharan schoolteachers may play a key role in the oral health promotion of primary schoolchildren and particularly for the prevention of HIV/AIDS-related oral health problems. Accordingly, the specific objectives of the study were: 1) to examine the level of knowledge about oral disease and the prevention of HIV/AIDS oral lesions amongst schoolteachers of Tanzania and Burkina Faso; 2) to identify their sources of information; 3) to evaluate their ability for HIV/AIDS intervention; and finally 4) to judge whether support by health staff is needed for oral health education in schools.

## Study Population and Methods

This study included two countries of Western and Eastern Africa, i.e. Burkina Faso and the United Republic of Tanzania. The key country estimates of the HIV epidemic in 2019 was reported by UNAIDS.^20^ For Burkina Faso, the national HIV prevalence rate at ages 15–49 years was 0.7%, the number of children aged 0-14 living with HIV was 9800, and the number of new HIV infections (0–14 years) was 880. The corresponding figures for Tanzania were: HIV prevalence rate (15–49 years) of 4.8%, the number of children aged 0-14 living with HIV was 93,000, while the number of new infections (0–14 years) amounted to 8600.

The present study was part of a comprehensive project on oral health related to HIV/AIDS in Africa, which was supported by the University of Copenhagen and the Ministry of Foreign Affairs of Denmark (Danida). The study received support from the national health research authorities. The multi-centre study was implemented as a cross-sectional study in the two Sub-Saharan African countries. Participants consisted of primary school teachers selected by stratified cluster sampling, i.e. 261 teachers from Tanzania and 313 teachers from Burkina Faso. In Tanzania, 51.9% of the teachers were from the capital of Dar es Salam, 25.2% from centrally located Iringa and 22.9% from Mbeya, which is located in the southwestern part of the country and the first urban settlement if travelling from Zambia. In Burkina Faso, 41.5% of the teachers were recruited from the capital Ouagadougou, while 58.5% of the teachers were from the second largest town, Bobo-Dioulasso.

All participants were invited to complete a structured questionnaire prepared for self-administration. The questions were designed from a standardised questionnaire developed by the WHO,^28^ which was validated in similar surveys. The final questionnaire included the following variables: 1) general health of children; 2) assessment of oral health status of schoolchildren and the estimated need for treatment; 3) causes of tooth decay, bleeding gums, and halitosis; 4) disease prevention; 5) awareness of the causes of HIV/AIDS; 6) signs of infection in general and symptoms of the mouth; 7) consequences of HIV/AIDS; 8) intervention against HIV/AIDS undertaken by parents and schools; 9) health activities carried out by schoolteachers; 10) children actually affected by HIV/AIDS and treatment strategies; 11) role of schoolteachers in oral health promotion, and 12) ability to address oral health issues in the classroom. The questionnaire in English was translated into Kiswahili and French; the items were carefully discussed and adapted to the local conditions and then pre-tested among schoolteachers not included in the final study.

Data entry and statistical evaluation were performed by means of SPSS (version 25; Armonk, NY, USA). Uni- and bivariate frequency distributions were used to analyse the data, and differences between proportions were examined using Chi^2^ tests.

## Results

### Oral Health

Overall, 7.7% of schoolteachers were of the opinion that children from their class had poor teeth and mouth, 58.7% reported that the oral health of students was fair, while 24.7% estimated the oral health of their students to be good or very good. Moreover, 27.3% of teachers assumed that their students needed substantial treatment, whereas 35.2% of teachers considered that children were in need of some dental treatment.

Regarding awareness about causes of dental decay, 79.1% of all teachers emphasised the importance of bacteria, 59.9% of teachers mentioned the role of sugars and 45.5% indicated unhealthy diet being key risk factors for dental caries. Meanwhile, 30.8% also reported that dental caries may be due to virus infection. As to principal measures for disease prevention, schoolteachers mentioned the significance of toothbrushing (86.9%), avoiding sugars (51.7%), consumption of healthy diet (30.1%), seeing a dentist (59.2%), and the use of fluoride (50.4%). It is noteworthy that only minor differences in dental knowledge were found between schoolteachers from Tanzania and Burkina Faso. However, 71.5% of teachers from Burkina Faso specified the use of fluoride as an important caries preventive action, while this action was mentioned by only 25.3% of the Tanzanian teachers (p < 0.001).

As to bleeding gums, bacteria (47.4%), neglect of toothbrushing (55.0%), incorrect toothbrushing (55.9%) and unhealthy diet (35.8%) were the factors mentioned most frequently by teachers. In addition, teachers suggested general illness (25.2%), hereditary factors (9.2%), and virus infection (7.5%). As to preventive actions, seeing a dentist was indicated by 78.0% of all teachers, while 56.3% of the teachers stated proper toothbrushing. It is worth mentioning that 46.0% of Burkinabe teachers saw the use of fluoride as a protective means for bleeding gums, vs 24.1% of teachers from Tanzania who did so (p < 0.001).

Finally, several of the teachers related bad mouth odour to lack of toothbrushing (76.6%), food remaining between teeth (75.6%), bacteria (72.8%), and general illness (26.8%), whereas 8.6% mentioned the role of viruses. Toothbrushing (85.0%), use of mouthwashes (54.9%), brushing the tongue regularly (47.5%), and rinsing the mouth with water (20.0%) were suggested actions for the prevention of oral malodour.

### HIV/AIDS

The majority of all teachers (95.6%) answered that they were aware of HIV/AIDS and 92.3% stated a virus to be the direct cause of AIDS. The routes of infection stated most frequently were blood (92.3%), vertical transmission from mother to child (85.4%) and sexual relations (68.4%). Young persons were considered vulnerable to HIV/AIDS by 82.4% of the Tanzanian and by 65.6% of the Burkinabe teachers (p < 0.001). In all, sex workers (70.3%) and long-distance drivers (46.2%) were other population groups identified to be at high risk. As indicated by Table 1, teachers were well aware of the general symptoms of HIV/AIDS, while the oral symptoms were reported less often.

**Table 1:** Percentages of schoolteachers in Burkina Faso and Tanzania stating certain general and oral signs of HIV/AIDS

	Burkina Faso	Tanzania	Total
**Important general signs of HIV/AIDS**			
General illness	73.6**	17.2	47.7
Fever	46.5	59.8*	52.5
Cough	61.5	78.2*	69.1
Weight loss	91.2**	66.7	80.0
Skin affected	81.6**	64.8	73.9
**Important signs of HIV/AIDS in the oral cavity**			
Pain	17.3	15.7	16.6
Dry mouth	35.8	51.7*	43.0
Difficulty eating	31.1	45.2*	37.5
Bad breath	16.6	19.9	18.1
Bad taste	16.3	35.2**	24.9
Bleeding from gums/mouth	27.9	23.8	26.0

*p < 0.01, **p < 0.001

In total, 17.6% of schoolteachers reported that in their classes, some children suffered from HIV/AIDS, while 3.8% reported that some children had been tested. Likewise, 10.3% of the teachers knew of students receiving ART, 3.1% claimed that ART is not affordable, and 2.4% pointed out that ART was not available in the community.

### Sources of Information

Television (81.9%), books (70.8%), radio (67.2%), and dentists (64.8%) were the most frequent replies to the question about sources of information concerning teeth and mouth among schoolteachers; no statistically significant differences were found between countries. Table 2 illustrates the sources of information about HIV/AIDS; awareness of the disease was received mainly through mass media, while teaching colleagues and health personnel played a somewhat smaller role in communication. Only a few of the schoolteachers answered that they received information about HIV/AIDS from a dentist. Of note, 67.6% of schoolteachers were of the opinion that a dentist must play an important role against the spread of HIV.

**Table 2:** Sources of information on HIV/AIDS of schoolteachers in Burkina Faso and Tanzania (%)

Source of information	Burkina Faso	Tanzania	Total
TV/radio	90.9	91.2	91.0
Newspaper/magazine	77.8	78.5	78.1
Other school teachers	47.9*	29.5	39.5
Other health personnel (e.g. hospital)	38.2	37.9	38.1
Medical doctor	42.3*	27.6	35.6
Key person in the community	34.9	31.8	33.5
Others	33.2	21.5	27.8
Relatives	20.2	26.1	22.9
Primary health worker	21.3	21.1	21.2
Mother	11.5	16.9	14.0
Father	10.9	16.1	13.3
Sister/brother	13.5	8.8	11.3
Dentist	7.7	3.8	5.9
Grandparents	4.5	3.8	4.2

*p < 0.001.

### Schoolteachers’ Role in Oral Health Education

In all, 61.5% of teachers had taught about oral health over the previous academic year. This activity primarily covered tooth cleaning (55.2%), the importance of dental health (44.9%), teeth and mouth (36.5%), sugars and oral health (34.6%), unhealthy foods and drinks (28.0%), and healthy diet (22.1%). Schoolteachers from Tanzania (70.5%) were more often engaged in classroom-based oral health education than were the Burkinabe teachers (53.9%) (p < 0.001). Similarly, 21.1% of Tanzanian schoolteachers informed that they arranged annual health meetings for parents or relatives of the children, whereas such meetings were organised by only 4.1% of teachers in Burkina Faso (p < 0.001).

Problems related to HIV/AIDS and the mouth were explained to children by 15.0% of all teachers; the Tanzanian schoolteachers mentioned this activity more often (20.3%) than did the Burkinabe teachers (10.5%) (p < 0.01). In total, 83.2% of schoolteachers declared that they should ensure teaching children about HIV/AIDS and the mouth. Furthermore, 89.1% agreed with the statement that teachers should teach children about the causes of dental caries and bleeding gums, 86.6% affirmed that teachers should advise about diet and sugar, and finally, 90.7% recognised that schoolteachers should instruct children in how to personally take care of their own teeth.

Table 3 illustrates the ability of schoolteachers to engage in child oral health education. Nearly two-thirds (61.8%) of all schoolteachers stated that they were either satisfied or very satisfied with teaching children about oral health, and the difference between countries was only minor. However, Tanzanian schoolteachers were dissatisfied with their teaching input more often than were the Burkinabe teachers. When asked about their specific participation in oral health promotion and disease prevention. only 23.0% of the Burkinabe teachers considered their competence as good or very good, while this was the case for 53.1% of the Tanzanian teachers (p < 0.001). Likewise, 18.3% of the Burkinabe teachers vs 48.3% of the Tanzanian teachers reported that they had little or very little support from health staff when organising oral health interventions at school (p < 0.001).

Table 3Schoolteachers’ responses regarding their involvement in oral health interventions at school (%)

**a.** Satisfaction with being involved in oral health education for children

	Burkina Faso	Tanzania	Total
Very dissatisfied /dissatisfied	13.1	33.4*	22.4
Satisfied	40.7	41.8	41.2
Very satisfied	24.0	16.5	20.6
Don’t know	22.2	8.4	15.9

*p < 0.001

**b.** In terms of having sufficient knowledge, how would you rate your competence for performing oral health education at school?

	Burkina Faso	Tanzania	Total
Very poor	11.5	1.9	7.1
Poor	27.8	13.4	21.3
Fairly good	31.3	27.2	29.4
Good	16.6	34.1*	24.6
Very good	6.4	16.9*	11.1
Don’t know	6.4	6.5	6.4

*p < 0.001

**c.** Support received from health staff for performing oral health education at school

	Burkina Faso	Tanzania	Total
Very little	15.1	28.4*	21.1
Little	3.2	19.9*	10.8
Some help	1.3	27.6*	13.3
A lot	1.3	2.7	1.9
Don’t know	79.2	21.5	52.9

*p < 0.001

## Discussion

The project was implemented in key low-income countries of the Sub-Saharan region. The participants were sampled from major urban centres, mainly to ensure the enrolment of a sufficient number of PLWHAs in the study. Selection of schools was done by cluster sampling through assistance from HIV/AIDS clinics and the NGOs supporting PLWHA organisations found in the areas selected. Although the sample of this study might not be representative for schoolteachers in purely statistical terms, it provides a critical picture of the situation among urban primary school teachers in low-income Sub-Saharan countries. Data were collected by means of structured questionnaires for self-completion. Having a number of alternate answers to choose from may perhaps result in unusual attention. Over-reporting of socially acceptable behaviour and attitudes is yet another limitation.

The study sought to explore whether primary schoolteachers can play a role in improving the oral health-related quality of life of schoolchildren, especially regarding problems related to HIV/AIDS. Thus, the questionnaire focussed on oral health troubles, awareness of oral manifestations of HIV/AIDS, and on how teachers may contribute to oral health promotion activities in primary schools. The evaluation of problems associated with oral diseases among schoolchildren seems realistic. The principal causes of oral diseases were largely correctly identified by participants, particularly when considering the role of bacteria and consumption of sugars. However, in Tanzania several schoolteachers also indicated mistakenly that dental caries was related to viruses. While many schoolteachers in both countries pointed out the general symptoms of HIV/AIDS, they seemed to be less familiar with the oral manifestations. This pattern is in agreement with results obtained in a study carried out in Lesotho, which showed somewhat unreliable dental knowledge of nursing staff.^24^ Likewise, a survey conducted in Lesotho suggested that oral health personnel, dental nurses and community workers would benefit from further training about diagnosis and management of oral lesions of HIV.^19^

Knowledge of measures to prevent HIV/AIDS was somewhat less than schoolteachers’ awareness of mechanisms for public health intervention. Moreover, the majority of schoolteachers emphasised the significance of regular toothbrushing. The importance of oral hygiene was rated higher among schoolteachers of Burkina Faso, while the Tanzanian schoolteachers tended to emphasise avoiding sugars. In both countries, knowledge about the caries preventive effect of fluoride seemed unclear, as the study revealed some misunderstandings about a possible role of fluorides in gingival health. It leaves the impression of “layman’s knowledge” and mirrors the fact that teachers mainly received their information on oral health from mass media rather than from professional sources.

Most teachers taught about oral health during the recent school year, and they expressed a positive attitude towards oral health being promoted through the schools. Remarkably, Tanzanian teachers frequently considered their ability to teach about oral health as very good, while such confidence was somewhat lower among teachers from Burkina Faso. Participation in communication about HIV/AIDS and oral health was higher among Tanzanian teachers than among Burkinabe teachers, which likely reflects the difference in the HIV/AIDS burden between the two countries.^20,23^

This study indicated that schoolteachers have a positive attitude towards incorporating oral health into their overall teaching; however, they may need additional support from health professionals to do so. Continuing education is rare in LMICs of Africa, and teachers may therefore profit from technical assistance by the staff of the local health services. Notably, oral health education related to HIV/AIDS may involve uncomfortable conversations about sensitive or taboo topics or communicating to children of the opposite sex.^1^ Thus, linking schools, HIV/AIDS programmes, and people with technical knowledge about oral health may increase the development of high-quality oral health activities by schools.

Oral health systems are inadequate in most LMICs of Africa.^17^ With no public oral health promotion and disease prevention programmes, alternative approaches need to be identified for such activities. The majority of the world’s children now live in countries with gender parity at the primary school level and with high gross enrolment rates.^21^ Although the majority of Sub-Saharan countries still lag behind the global mean, the primary school is still the channel most likely to reach school-aged children. Given the negative impact that HIV/AIDS-related oral lesions have on the daily life of PLWHA, the reinforcement of good oral health, integrated disease prevention, proper self-care, and treatment of poor oral health is of great importance.

## Conclusion

Schoolteachers have the potential to advance the quality of life among schoolchildren by approaching HIV/AIDS through oral health. The present study confirms that schoolteachers may make relevant contributions to the fight against HIV/AIDS amongst African children, and that a teacher may play an essential role in oral health education, creation of healthy school environments, and the identification of children at risk of disease. The vast majority of schoolteachers declared that they are dedicated performing preventive work in schools, yet may need additional technical support from health personnel.
